# FiMAP: A fast identity-by-descent mapping test for biobank-scale cohorts

**DOI:** 10.1371/journal.pgen.1011057

**Published:** 2023-12-01

**Authors:** Han Chen, Ardalan Naseri, Degui Zhi

**Affiliations:** 1 Human Genetics Center, Department of Epidemiology, School of Public Health, The University of Texas Health Science Center at Houston, Houston, Texas, United States of America; 2 Center for Artificial Intelligence and Genome Informatics, McWilliams School of Biomedical Informatics, The University of Texas Health Science Center at Houston, Houston, Texas, United States of America; Emory University, UNITED STATES

## Abstract

Although genome-wide association studies (GWAS) have identified tens of thousands of genetic loci, the genetic architecture is still not fully understood for many complex traits. Most GWAS and sequencing association studies have focused on single nucleotide polymorphisms or copy number variations, including common and rare genetic variants. However, phased haplotype information is often ignored in GWAS or variant set tests for rare variants. Here we leverage the identity-by-descent (IBD) segments inferred from a random projection-based IBD detection algorithm in the mapping of genetic associations with complex traits, to develop a computationally efficient statistical test for IBD mapping in biobank-scale cohorts. We used sparse linear algebra and random matrix algorithms to speed up the computation, and a genome-wide IBD mapping scan of more than 400,000 samples finished within a few hours. Simulation studies showed that our new method had well-controlled type I error rates under the null hypothesis of no genetic association in large biobank-scale cohorts, and outperformed traditional GWAS single-variant tests when the causal variants were untyped and rare, or in the presence of haplotype effects. We also applied our method to IBD mapping of six anthropometric traits using the UK Biobank data and identified a total of 3,442 associations, 2,131 (62%) of which remained significant after conditioning on suggestive tag variants in the ± 3 centimorgan flanking regions from GWAS.

## Author summary

Identity-by-descent (IBD) segments shared from a common ancestor can be used in association mapping to identify genomic regions associated with complex traits such as height, weight and body fat distribution. In this work, we developed FiMAP, an efficient IBD mapping test that can be applied to hundreds of thousands of individuals from biobank-scale cohorts, by leveraging IBD segments from recent efficient IBD detection software programs. Built upon the classical variance component model which has its deep root in linkage analysis, FiMAP utilizes sparse linear algebra and random matrix algorithms to speed up the computation in large samples. We demonstrated accuracy, type I error control and statistical power of the FiMAP algorithm, and illustrated how IBD mapping using FiMAP could identify associations complementary to genome-wide association study findings. With the availability of an abundance of genetic data from large biobank-scale cohorts, FiMAP provides a unique angle to leverage such rich resources and better understand the genetic architecture of complex traits.

## Introduction

Identity-by-descent (IBD) segments between two individuals are inherited from their common ancestor, without recombination [1]. They have been widely used in forensic genetics [2,3], as well as population genetics to detect evidence of natural selection, and to estimate the demographic history such as bottlenecks and admixture [4–6]. In a genome-wide association study (GWAS) with cryptically related samples, the average proportion of the genome shared IBD can be used to infer the degree of relatedness [1,7], and to construct an empirical kinship matrix to account for cryptic relatedness in GWAS [8–11].

IBD mapping is the study of association between the sharing of IBD segments and phenotypic similarities. Early IBD mapping studies were mostly in linkage analysis for family studies [12–15]. IBD mapping methods for other study designs have also been developed, such as those searching for chromosome segments shared by distantly related patients [16], testing for variance components-based association with haplotype clusters [17], comparing pairwise IBD rates in case-case and case-control pairs [18], and testing for the variance component of the multiple-IBD cluster random effects for quantitative traits [19]. Indeed, IBD mapping has successfully identified genomic regions associated with Parkinson’s disease [20], serum triglycerides [21], multiple sclerosis [22], and amyotrophic lateral sclerosis [23].

Although IBD mapping is not yet a widely-applied method, it holds promise to uncover untyped rare variant and haplotypic effects, that are still escaping the search of the “missing heritability”. While most common variants are tagged by genotypes from arrays and can be well-imputed, rare variants are still not well covered. IBD mapping can indirectly test the effects of rare variants co-segregated with the IBD segments and recover the association signal. Further, the combination of multiple variants on an IBD segment, each individually weakly associated with the phenotype and thus difficult to be identified by single variant association tests, can be captured by IBD mapping. As the phase information is often ignored in traditional GWAS approaches and variant set tests for rare genetic variants [24–31], IBD mapping leverages such information and is less susceptible to genotyping or sequencing errors. It can better identify association signals from rare variants and haplotype effects [18], and therefore offers a unique angle to investigate genetic associations.

While promising, IBD mapping were not popular due to several technical challenges. First, IBD mapping requires accurate phasing of haplotypes. This difficulty is mostly addressed by current phasing software [32–34]. Second, the power of IBD mapping methods is linked with the number of IBD segments in the sample. While the density of IBD segments in families and inbred populations are high, their density in outbred populations can be much lower. Nonetheless, with the availabilities of large biobanks, the sample sizes are large enough to harbor a large number of IBD segments. For example, our recent studies found that each haplotype of a UK Biobank participant is covered by about ten 5 centimorgan (cM) IBD segments shared with other UK Biobank participants [35]. Third, traditional IBD segment calling methods are not efficient and cannot scale up to modern biobank-scale cohorts with hundreds of thousands of samples. Fortunately, this challenge is largely resolved by a new generation of efficient IBD segment detection methods, either based on the positional Burrows Wheeler transform (PBWT) algorithm [36], such as RaPID [37], hap-IBD [38], and TPBWT [39], or based on advanced string hashing such as FastSMC [40] and iLASH [41]. These methods typically achieve *O*(*N*) complexity, where *N* is the sample size, and have made IBD segment calling computationally feasible in biobank-scale cohorts with hundreds of thousands to millions of individuals. However, even with the advancements of abovementioned technologies, existing IBD mapping methods are still not scalable to large sample sizes due to lack of efficient statistical tests for IBD mapping.

**Fig 1:**
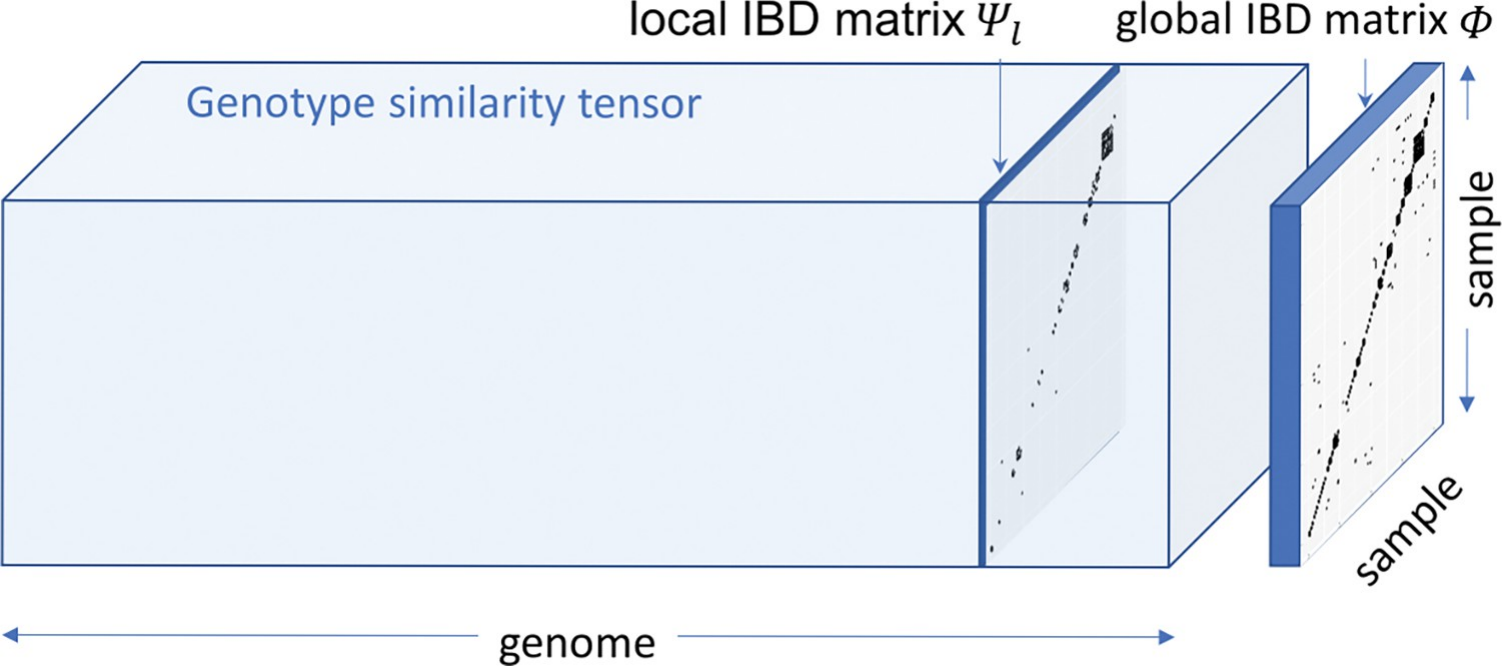
Local and global IBD matrices in FiMAP. The global IBD matrix *Φ* is the average of the local IBD matrices *Ψ*_*l*_ along the genome dimension of the genotype similarity tensor.

To address the main computational challenge for biobank-scale cohorts that any IBD mapping methods with *O*(*N*^2^) computational time complexity or higher would quickly become infeasible, we have developed FiMAP, a fast IBD mapping test for biobank-scale cohorts. FiMAP leverages IBD segments identified by IBD callers such as RaPID [37] and hap-IBD [38], and constructs a genotype similarity tensor on the whole genome (**Fig 1**). The genotype similarity tensor is a collection of *N*×*N* pairwise local IBD matrices that change across the whole genome. Each local IBD matrix is a sparse matrix indicating the proportion of IBD sharing between any two individuals in the given genomic region, with most elements being 0. The average of local IBD matrices across the whole genome is the global IBD matrix representing the average proportion of IBD sharing, which can be used as a kinship coefficient matrix to account for genetic relatedness in the study samples. FiMAP leverages a random matrix-based algorithm to speed up the computation, so that a genome-wide IBD mapping analysis for hundreds of thousands of study samples can be finished within a few hours. We also demonstrate the application of FiMAP to the IBD mapping analysis of six anthropometric traits in the UK Biobank.

## Description of the method

### Variance component models

Variance component models have long been used in linkage analysis to identify genetic loci linked with quantitative traits [12–15]. Consider a linear mixed model *Y*_*i*_ = *X*_*i*_*β*+*b*_*i*_+*δ*_*il*_+*ε*_*i*_ for individual *i*, where *Y*_*i*_ is the phenotype, *X*_*i*_ are *c* fixed-effect covariates with effect sizes *β*, *b*_*i*_ is the polygenic random effect accounting for global relatedness, *δ*_*il*_ is the random effect of local relatedness at genetic locus *l*, we stack *N* individuals to get length *N* vectors *Y*, *b*, *δ*_*l*_, *ε*, and an *N*×*c* matrix *X*, from *Y*_*i*_, *b*_*i*_, *δ*_*il*_, *ε*_*i*_, *X*_*i*_, respectively. The vector $b∼N(0,\sigma_{G}^{2}\Phi )$ are the random effects for the *N*×*N* global IBD (or kinship) matrix *Φ* denoting the average copies of the genome shared IBD between any two individuals, $\delta_{l}∼N(0,\sigma_{l}^{2}\Psi_{l})$ are the random effects for the *N*×*N* local IBD matrix *Ψ*_*l*_, defined as the copies of the genome shared IBD at this genetic locus *l*, and *ε* are independent and identically distributed errors that follow a normal distribution with mean 0 and variance $\sigma_{E}^{2}$. Under the null hypothesis of no association with the quantitative trait at genetic locus *l*, the random effects *δ*_*l*_ are equal to 0 and this is equivalent to testing variance component hypotheses $H_{0}:\sigma_{l}^{2}=0$ vs $H_{1}:\sigma_{l}^{2}>0$.

### The asymptotic test

Let $\hat{\Sigma }={\hat{\sigma }}_{E}^{2}I_{N}+{\hat{\sigma }}_{G}^{2}\Phi$, where both ${\hat{\sigma }}_{E}^{2}$ and ${\hat{\sigma }}_{G}^{2}$ are estimated from the null model (with *δ*_*l*_ = 0) in the matrix-vector form *Y* = *Xβ*+*b*+*ε*. Let $r=Y-X\hat{\beta }-\hat{b}$ be the length *N* residual vector, where both $\hat{\beta }$ and $\hat{b}$ are estimated from this null model, the classical score-type variance component test $Q_{l}=\frac{1}{{\hat{\sigma }}_{E}^{4}}r^{T}\Psi_{l}r$ asymptotically follows a chi-square mixture distribution ∑_*j*_*ζ*_*j*_*χ*_1,*j*_^2^ [28,42,43], where *χ*_1,*j*_^2^ are independent chi-square distributions with 1 degree of freedom, and *ζ*_*j*_’s are the eigenvalues of ${\hat{P}}^{\frac{1}{2}}\Psi_{l}{\hat{P}}^{\frac{1}{2}}$, where $\hat{P}={\hat{\Sigma }}^{-1}-{\hat{\Sigma }}^{-1}X(X^{T}{\hat{\Sigma }}^{-1}X{)}^{-1}X^{T}{\hat{\Sigma }}^{-1}$ is the *N*×*N* projection matrix [44–47].

### The finite-sample adjustment

Although under regularity conditions, ${\hat{\sigma }}_{E}^{2}$ and ${\hat{\sigma }}_{G}^{2}$ are consistent estimators for variance component parameters $\sigma_{E}^{2}$ and $\sigma_{G}^{2}$ under the null hypothesis $H_{0}:\sigma_{l}^{2}=0$, the classical score-type variance component test above treats them as fixed numbers and ignores the variability in their estimation, which could result in not well-calibrated p values in finite samples. This is a known issue for score-type variance component tests in microbiome association studies with small sample sizes [48–50]. Despite large sample sizes in biobank-scale cohorts, the local IBD matrix *Ψ*_*l*_ for genetic locus *l* is often sparse, which could invalidate asymptotic inference on the quadratic form $Q_{l}=\frac{1}{{\hat{\sigma }}_{E}^{4}}r^{T}\Psi_{l}r$. We note that ${\hat{\Sigma }}^{-1}(Y-X\hat{\beta })={\hat{\sigma }}_{E}^{-2}r$, and ${\hat{\sigma }}_{E}^{2}=\frac{{\left(Y-X\hat{\beta }\right)}^{T}{\left(\frac{\hat{\Sigma }}{{\hat{\sigma }}_{E}^{2}}\right)}^{-1}(Y-X\hat{\beta })}{N-c}=\frac{r^{T}\hat{\Sigma }r}{{\hat{\sigma }}_{E}^{2}(N-c)}$, where $\frac{\hat{\Sigma }}{{\hat{\sigma }}_{E}^{2}}$ is free of $\sigma_{E}^{2}$. To account for the variability in estimating $\sigma_{E}^{2}$, we can rewrite $Q_{l}=(N-c)\frac{r^{T}\Psi_{l}r}{r^{T}\hat{\Sigma }r}$, and compute the finite-sample p-value as *P*(∑_*j*_*ξ*_*j*_*χ*_1,*j*_^2^>0), where *ξ*_*j*_’s are the eigenvalues of ${\hat{P}}^{\frac{1}{2}}{(\Psi }_{l}-\frac{Q_{l}}{N-c}\hat{\Sigma }){\hat{P}}^{\frac{1}{2}}$.

### FiMAP

As both $\hat{P}$ and *Ψ*_*l*_ are *N*×*N* matrices (and $\hat{P}$ is not sparse), conducting the classical score-type variance component test, without or with the finite-sample adjustment, requires *O*(*N*^2^) memory footprint and *O*(*N*^3^) computational time complexity, which becomes infeasible for hundreds of thousands to millions of individuals. To solve this computational challenge, we use an *N*×*B* random matrix *R* to approximate the eigenvalues of ${\hat{P}}^{\frac{1}{2}}\Psi_{l}{\hat{P}}^{\frac{1}{2}}$ by performing eigen decomposition of a *B*×*B* matrix $\frac{1}{B}R^{T}\Psi_{l}R$. Similar random matrix algorithms have previously been applied to principal component analysis [51,52], heritability [53] and genetic correlation [54] estimation, dynamic scanning of rare variant associations [45,55], and stochastic summary statistics [56]. Specifically, we start with a Cholesky decomposition of the global IBD matrix *Φ* = *LL*^*T*^, then simulate length *N* random vectors *r*_1_ and *r*_2_ from a standard normal distribution to compute $r_{3}={\hat{\sigma }}_{E}r_{1}+{\hat{\sigma }}_{G}Lr_{2}$ and $r_{4}={\hat{\Sigma }}^{-1}r_{3}-{\hat{\Sigma }}^{-1}X(X^{T}{\hat{\Sigma }}^{-1}X{)}^{-1}X^{T}{\hat{\Sigma }}^{-1}r_{3}$. Therefore, we have $r_{4}∼N(0,\hat{P})$ and repeat the process *B* times to get an *N*×*B* random matrix *R*. In reality, the global IBD matrix *Φ* often has non-zero but small off-diagonal elements and we can set them to zero to ensure that it is block-diagonal (with a bounded largest block size) and sparse, then both *L* and ${\hat{\Sigma }}^{-1}$ are also block-diagonal, and *r*_3_ and *r*_4_ do not require any matrix-vector multiplications involving full dense *N*×*N* matrices. If both the largest block size and *B* are *O*(1), then the overall computational complexity of FiMAP is *O*(*N*). Moreover, for the finite-sample adjustment, we can pre-compute $\frac{1}{B}R^{T}\hat{\Sigma }R$ only once in a genome-wide scan, and approximate the eigenvalues of ${\hat{P}}^{\frac{1}{2}}{(\Psi }_{l}-\frac{Q_{l}}{N-c}\hat{\Sigma }){\hat{P}}^{\frac{1}{2}}$ by performing eigen decomposition of a *B*×*B* matrix $\frac{1}{B}R^{T}\Psi_{l}R-\frac{Q_{l}}{(N-c)B}R^{T}\hat{\Sigma }R$.

In reality, we observe the start and end positions of each IBD segment between two individuals. Therefore, instead of a local IBD matrix *Ψ*_*l*_ with 2 identical copies of the genome shared IBD at this genetic locus *l* for each individual with themselves, we use ${\tilde{\Psi }}_{l}=\Psi_{l}-2I_{N}$ in practice, with each element denoting the copies of the genome shared IBD between two individuals in the given region. For biobank-scale cohorts, ${\tilde{\Psi }}_{l}$ is highly sparse with most elements 0. Assuming a total of *L* equally spaced genomic regions across the whole genome, the global IBD matrix $\Phi =\frac{1}{L}\sum_{l=1}^{L}{\tilde{\Psi }}_{l}+2I_{N}$ is the average of the local IBD matrices *Ψ*_*l*_’s. In our implementation, we pre-compute an offset $A=2R^{T}R-\frac{2r^{T}r}{N-c}R^{T}\hat{\Sigma }R$ only once in a genome-wide scan. For each genetic locus *l*, we then compute the finite-sample p-value as $P(\sum_{j}{\tilde{\xi }}_{j}{\chi_{1,j}}^{2}>0)$, where ${\tilde{\xi }}_{j}$’s are the eigenvalues of $\frac{1}{B}\{A+R^{T}{\tilde{\Psi }}_{l}R-\frac{r^{T}{\tilde{\Psi }}_{l}r}{N-c}R^{T}\hat{\Sigma }R\}$. Note that $\frac{1}{B}R^{T}\hat{\Sigma }R$ is also pre-computed, for each genetic locus *l* with an observed local IBD matrix ${\tilde{\Psi }}_{l}$, we only need to update the *B*×*B* matrix $R^{T}{\tilde{\Psi }}_{l}R$ and the scalar $r^{T}{\tilde{\Psi }}_{l}r$.

## Verification and comparison

### Asymptotic and finite-sample p values

We used RaPID and hap-IBD IBD segment calls from the UK Biobank array-typed genotype data to simulate phenotype data, and evaluated type I error rates and power of FiMAP in identifying genetic associations. This genotype dataset consists of 658,720 variants on 22 autosomes. Specifically, RaPID and hap-IBD IBD segments were called using the phased haplotypes released by the UK Biobank team [57], on all 22 autosomes to construct the global IBD matrix *Φ* for 487,252 individuals. For individuals with no inbreeding, the diagonal elements of *Φ* is equal to 2, which is 4 times the theoretical kinship coefficient, indicating the total copies of the genome shared IBD by each individual with themselves. The off-diagonal elements of *Φ* are the average copies of the genome shared IBD by each pair of individuals. To ensure the sparsity of the global IBD matrix *Φ*, we set off-diagonal elements less than 0.088 to 0, which is equivalent to including fourth-degree relatives or closer in the kinship matrix (kinship coefficient ≥ 0.022). We first compared the performance of asymptotic and finite-sample variance component tests, using either the dense *N*×*N* projection matrix $\hat{P}$ estimated from the null model or the FiMAP algorithm with the number of random vectors *B* = 100, 1,000 or 10,000. Given the *O*(*N*^3^) complexity and *O*(*N*^2^) memory footprint to directly use the dense *N*×*N* projection matrix $\hat{P}$, instead of using the full UK Biobank samples, we took a random subset of *N* = 10,000 samples and fitted a null linear mixed model for waist circumference adjusting for age, age^2^, sex, their interactions, and top 10 ancestral principal components (PCs), and used the global IBD matrix to model the covariance structure of the random intercept. We then simulated random *N*×*N* symmetric local IBD matrices with 10,000, 100,000 and 1 million non-zero off-diagonal elements from a uniform distribution between 0 and 2. These matrices were not associated with waist circumference. We evaluated the p value calibration of asymptotic and finite-sample tests, as well as the accuracy of approximation in the FiMAP algorithm.

**S1** and **S2 Figs** show simulation results using a random subset of *N* = 10,000 samples from the UK Biobank, under the null hypothesis of no genetic association with waist circumference. Finite-sample p values from both the FiMAP algorithm and directly using dense $\hat{P}$ were well calibrated with 10,000, 100,000 and 1 million non-zero off-diagonal elements in the simulated local IBD matrices (**S1A, S1B and S1C Fig**). However, asymptotic p values were not well calibrated if the number of random vectors *B* used in the FiMAP algorithm was small, or the number of non-zero off-diagonal elements in the simulated local IBD matrices was small (**S1D, S1E and S1F Fig**). Of note, with 10,000 non-zero off-diagonal elements in the simulated local IBD matrices, even the asymptotic p values from directly using dense $\hat{P}$ did not follow a uniform distribution under the null hypothesis. **S2A, S2B and S2C Fig** show that finite-sample FiMAP p values were highly concordant with finite-sample variance component test p values using dense $\hat{P}$, regardless of the sparsity of local IBD matrices. On the other hand, asymptotic and finite-sample variance component test p values using dense $\hat{P}$ were quite different for sparse local IBD matrices (**S2D Fig**), but much closer as local IBD matrices became denser (**S2E and S2F Fig**). Compared to asymptotic variance component test p values using dense $\hat{P}$, asymptotic FiMAP p values got closer with an increasing *B* and/or denser local IBD matrices (**S2G, S2H and S2I Fig**). It is also worth noting that the asymptotic p values in **S2D, S2E and S2F Fig** were the same as the dense $\hat{P}$ p values in **S2G, S2H and S2I Fig**, respectively, and they only performed well with denser local IBD matrices (**S1D, S1E and S1F Fig**). Based on these results, we only presented finite-sample FiMAP p values with *B* = 100 in subsequent simulations and real data analysis.

### Type I error rates

For the full UK Biobank samples, we chunked 22 autosomes into 1 cM windows and assembled 3,403 local IBD matrices by computing the proportion of IBD sharing between each pair of individuals in each 1 cM window (the last window on each chromosome is shorter than 1 cM). In type I error simulations, we randomly selected 400,000 individuals and subset the global IBD matrix *Φ* to get a submatrix *Φ*_0_. We then simulated age from a normal distribution with mean 50 and standard deviation 5, and sex from a Bernoulli distribution with probability 0.5. A continuous phenotype *Y*_*i*_ for individual *i* was simulated as *Y*_*i*_ = 0.05*age*_*i*_+0.5*sex*_*i*_+*b*_*i*_+*ε*_*i*_, where the vector form *b* followed a multivariate normal distribution with mean 0 and covariance matrix *Φ*_0_, and *ε*_*i*_ followed a standard normal distribution. Then we tested for the association with 3,403 local IBD matrices after adjusting for age and sex. We simulated 50 phenotype replicates and obtained a total of 170,150 p values under the null hypothesis of no local IBD random effects.

**Fig 2:**
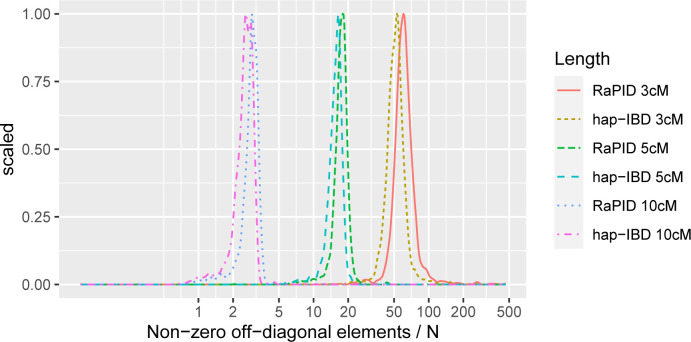
Distribution of average numbers of non-zero IBD sharing per individual in local IBD matrices. For each color, a total of 3,403 local IBD matrices of sample size N = 487,252 for 1 cM windows were plotted, with density estimates scaled to maximum of 1. The local IBD matrices were constructed from RaPID and hap-IBD IBD segments called with length ≥ 3 cM, 5 cM, 10 cM.

We counted the numbers of non-zero off-diagonal elements in each of the 3,403 local IBD matrices for 1 cM windows, with sample size N = 487,252. The local IBD matrices were constructed from RaPID and hap-IBD IBD segments called with length ≥ 3 cM, 5 cM, 10 cM. **Fig 2** shows that the number of non-zero off-diagonal elements increased as the IBD length cutoff decreased from 10 cM, 5 cM to 3 cM, for IBD segments called from both RaPID and hap-IBD. For example, when the RaPID IBD length cutoff was 3 cM, the median number of non-zero off-diagonal elements in local IBD matrices was 60.4 × N, with a range of 8.2 × N to 468.0 × N across locations. For hap-IBD IBD segments with length ≥ 3 cM, the median number of non-zero off-diagonal elements in local IBD matrices was 51.6 × N, with a range of 2.4 × N to 276.6 × N across locations. These results suggest that in the UK Biobank data, the largest number of non-zero off-diagonal elements in any local IBD matrix at a given length cutoff (≥ 3 cM, 5 cM, or 10 cM) was much less than what we would expect from a dense *N*×*N* matrix, regardless of the IBD caller, thus we could consider all these local IBD matrices as sparse and the computational efficiency of FiMAP was guaranteed. On a computing server with dual Intel Xeon E5-2687W v4 CPU (3.00 GHz, 24 cores in total), analysis of each simulation replicate with N = 400,000 took about 131 and 115 minutes for RaPID and hap-IBD IBD length cutoff 3 cM, 36 and 33 minutes for 5 cM, and 9.4 and 8.9 minutes for 10 cM, respectively, using 40 threads in parallel.

**Fig 3:**
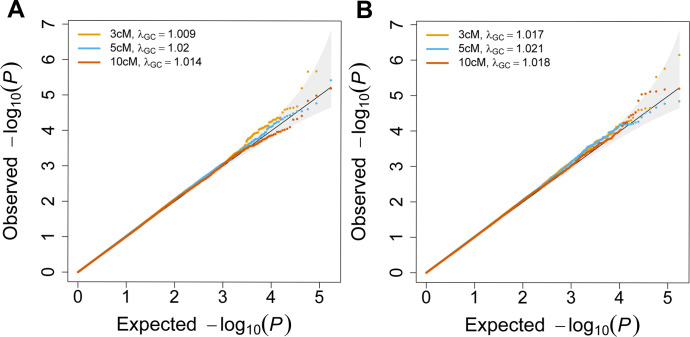
Quantile-quantile plot of finite-sample FiMAP p values under the null hypothesis. A total of 170,150 p values from 50 simulation replicates were plotted. The genomic inflation factor *λ*_*GC*_ was computed using the observed median p values. The local IBD matrices were constructed from (A) RaPID or (B) hap-IBD IBD segments called with length ≥ 3 cM, 5 cM, 10 cM.

Finite-sample FiMAP p values under the null hypothesis of no local IBD random effects were shown in **Fig 3**. FiMAP p values with the finite-sample adjustment were well-calibrated, for IBD segments called from both RaPID and hap-IBD. The median finite-sample FiMAP p value showed genomic inflation factors very close to 1 for IBD segments called with length ≥ 3 cM, 5 cM, 10 cM, suggesting that FiMAP with the finite-sample adjustment is a fast and valid IBD mapping test in large samples, regardless of the IBD caller.

### Power

We also conducted power simulations to benchmark finite-sample FiMAP results with standard GWAS results using the single-variant score test from GMMAT [44] which fitted exactly the same null model adjusting for age and sex, with a variance component parameter for the global IBD random effects. Using a random sample of N = 400,000 individuals from the UK Biobank, we simulated untyped ultra-rare causal variants that were not included in any of the tests for a fair comparison. Specifically, in each simulation replicate, we randomly selected a 1 cM window with at least 2 ultra-rare variants with MAF < 0.0005, we then assumed that all *J* ultra-rare variants with MAF < 0.0005 in this window were causal variants and simulated a continuous phenotype *Y*_*i*_ for individual *i* as $Y_{i}=0.05age_{i}+0.5sex_{i}+\sum_{j=1}^{J}G_{ij}\beta_{j}+b_{i}+\varepsilon_{i}$, where *age*_*i*_, *sex*_*i*_, *b*_*i*_ and *ε*_*i*_ were simulated using the same parameter settings as in the type I error simulations, and the causal variant effect $\beta_{j}=\sqrt{\frac{0.02}{2MAF_{j}(1-MAF_{j})}}$. Finite-sample FiMAP with local IBD matrices constructed from RaPID and hap-IBD IBD segments called with length ≥ 3 cM, 5 cM, 10 cM were conducted. Using the UK Biobank imputed genotype data with 89 million variants on 22 autosomes, we excluded variants with missing rate > 5% or MAF < 1% to mask the ultra-rare causal variants, and conducted a standard single-variant test commonly used in GWAS. We tested both the 1 cM window from which causal variants were simulated for statistical power, and the neighboring ± 1 cM windows for localization. Empirical power was estimated for FiMAP as the proportion of p values being less than the Bonferroni-corrected significance level of 0.05/3,403 = 1.47 × 10^−5^, and for GWAS as the proportion of any minimum single-variant p values in each 1 cM testing window being less than the genome-wide significance level of 5 × 10^−8^, over 1,000 simulation replicates.

**Fig 4:**
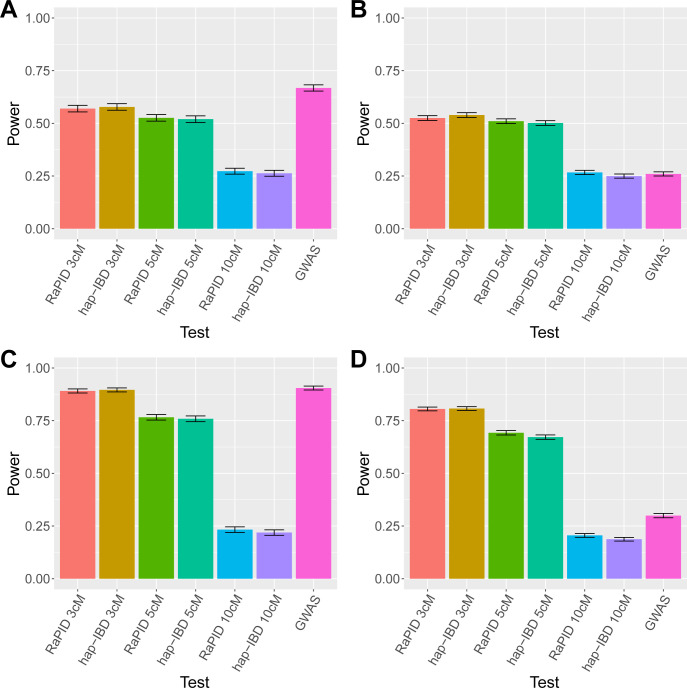
Power comparison of FiMAP and GWAS. (A) Untyped rare causal variants, 1 cM causal window; (B) Untyped rare causal variants, 1 cM neighboring windows; (C) Haplotype effects, 1 cM causal window; (D) Haplotype effects, 1 cM neighboring windows. In each simulation replicate, finite-sample FiMAP with local IBD matrices constructed from RaPID and hap-IBD IBD segments called with length ≥ 3 cM, 5 cM, 10 cM were benchmarked with a GWAS single-variant test. Empirical power was estimated for FiMAP as the proportion of finite-sample p values less than 0.05/3,403 = 1.47 × 10^−5^, and for GWAS as the proportion of any minimum single-variant p values in each 1 cM testing window less than 5 × 10^−8^, over 1,000 simulation replicates.

Assuming the causal variants in a 1 cM window were ultra-rare (with MAF < 0.0005) and not directly observed, **Fig 4A** shows that FiMAP had less power than the GWAS single-variant test on imputed variants. **Fig 4B** shows empirical power to identify the association in the neighboring ± 1 cM windows from the simulated untyped rare causal variants. Compared to **Fig 4A**, FiMAP power dropped slightly in these neighboring windows, while the GWAS single-variant test power plummeted more dramatically. This is not surprising because the IBD segments used in the FiMAP analysis were all longer than the 1 cM window, while for the GWAS single-variant test, the linkage disequilibrium decayed much faster in the neighboring windows, suggesting that FiMAP had a lower resolution than the GWAS single-variant test in association mapping.

In addition, we simulated haplotype effects and compared FiMAP with IBD segments called with length ≥ 3 cM, 5 cM, 10 cM with the GWAS single-variant test. Specifically, we estimated the start and end positions for all haplotypes with length ≥ 0.5 cM shared by at least 500 individuals in the UK Biobank using a PBWT-block algorithm [58]. In each simulation replicate, we used a random sample of N = 400,000 individuals and randomly sampled a causal haplotype from an autosome. We simulated a continuous phenotype *Y*_*i*_ for individual *i* as $Y_{i}=0.05age_{i}+0.5sex_{i}+H_{i}\gamma +b_{i}+\varepsilon_{i}$, where *age*_*i*_, *sex*_*i*_, *b*_*i*_ and *ε*_*i*_ were simulated using the same parameter settings as in the type I error simulations, and *H*_*i*_ was the number of causal haplotypes carried by individual *i*, with possible values 0, 1, 2. The causal haplotype effect was assigned as $\gamma =\sqrt{\frac{0.02}{Var(H_{i})}}$. We defined the “causal” window as: 1) for short causal haplotypes located completely within a 1 cM window, that 1 cM window; 2) for causal haplotypes that spanned two 1 cM windows, the window with a larger overlap with the causal haplotype; and 3) for causal haplotypes that spanned more than two 1 cM windows, the first window that was completely covered by the causal haplotype. We then tested the 1 cM “causal” window using FiMAP and GWAS single-variant test, with the same significance levels as above and 1,000 simulation replicates for statistical power. For localization, we tested two closest 1 cM windows that did not overlap with the simulated causal haplotype.

In the presence of causal haplotype effects, the GWAS single-variant test on imputed variants also had the best power, followed by FiMAP with IBD segments called with length ≥ 3 cM and 5 cM (**Fig 4C**). FiMAP with IBD segments called with length ≥ 10 cM suffered from a substantial power loss, likely due to the fact that many shorter IBD segments tagging the causal haplotype in the region were not included. **Fig 4D** shows empirical power to identify the association in the two closest 1 cM windows that did not overlap with the simulated causal haplotype, and we observed similar patterns as seen in **Fig 4B**.

Although in both power simulation settings, FiMAP did not outperform the GWAS single-variant test on densely imputed variants for empirical power, we compared proportions of simulation replicates in which both FiMAP and GWAS or only one approach identified the association. **Table 1** shows that while many causal windows were identified by both approaches, FiMAP and GWAS each identified some unique associations, suggesting that FiMAP might provide complementary association evidence missed by the classical GWAS approach. Therefore, we conducted FiMAP, GWAS, as well as conditional FiMAP analysis adjusting for GWAS tag variants for 6 anthropometric traits from the UK Biobank. Furthermore, **Fig 4** shows that in all simulation scenarios, FiMAP power was similar for IBD segments with the same length cutoff, for both RaPID and hap-IBD. Therefore, we present only FiMAP results from RaPID IBD segments with length ≥ 3 cM in the analysis of UK Biobank anthropometric traits.

**Table 1:** Proportions of power simulation replicates in which both FiMAP and GWAS or only one approach identified the association.

Scenario	Window	Both	FiMAP only	GWAS only
Untyped rare causal variants	Causal	38.6% (1.5%)	18.4% (1.2%)	28.2% (1.4%)
Untyped rare causal variants	Neighboring	11.1% (0.7%)	41.4% (1.1%)	14.9% (0.8%)
Haplotype effects	Causal	82.8% (1.2%)	6.3% (0.8%)	7.7% (0.8%)
Haplotype effects	Neighboring	28.2% (1.0%)	52.3% (1.1%)	1.7% (0.3%)

Finite-sample FiMAP with local IBD matrices constructed from RaPID IBD segments called with length ≥ 3 cM were benchmarked with a GWAS single-variant test on imputed genotypes. In each simulation replicate, association was defined as being identified by FiMAP if the finite-sample p value was less than 0.05/3,403 = 1.47 × 10^−5^, and by GWAS if the minimum single-variant p value in each 1 cM testing window was less than 5 × 10^−8^. Proportions of simulation replicates in which both FiMAP and GWAS, or FiMAP but not GWAS, or GWAS but not FiMAP identified the association were estimated using 1,000 replicates. Standard errors of these proportions were shown in parentheses.

### Applications

We applied FiMAP to 6 anthropometric traits: waist circumference (N = 407,872), hip circumference (N = 407,827), standing height (N = 407,681), sitting height (N = 407,323), body mass index (N = 407,255), and body weight (N = 407,400), from the UK Biobank white British study participants with genetic ethnic group in Caucasians, after excluding individuals with inconsistent gender and biological sex. Each trait was adjusted for age, age^2^, sex, their interactions, and top 10 ancestral PCs, and the residuals were rank normalized and analyzed using a linear mixed model with the same aforementioned fixed-effects covariates [59] and the global IBD matrix to model the covariance structure of the random intercept. We used RaPID IBD segment calls with length ≥ 3 cM, and defined genomic regions *l* using 1 cM windows as used in our simulation studies, with a total of 3,403 test regions on 22 autosomes. We set the number of random vectors used in FiMAP *B* = 100 in all UK Biobank data analyses. The summary statistics are presented in **S1–S6 Tables**. For each trait, genome-wide significance was defined as finite-sample FiMAP p value < 0.05/3,403 = 1.47 × 10^−5^, the Bonferroni-corrected threshold for 3,403 tests.

We also analyzed the same anthropometric traits in a GWAS setting using GMMAT [44] on 22 million imputed genetic variants with MAF ≥ 0.0001, imputation quality score ≥ 0.3, and missing rate < 5%. For each trait, suggestive associations were defined as single-variant test p values < 1 × 10^−6^. In the conditional analysis, for each 1 cM window with a significant finite-sample FiMAP p value < 1.47 × 10^−5^, we considered all imputed genetic variants with suggestive association evidence from the GWAS single-variant test in the ± 3 cM flanking regions (a total length of 7 cM) to account for the fact that the IBD segments we used had a length ≥ 3 cM. Specifically, we clumped these suggestive variants into 100kb windows and selected the most significant variant from each window to form a conditional set. We then adjusted for this set of variants as covariates in the conditional FiMAP analysis.

**Fig 5:**
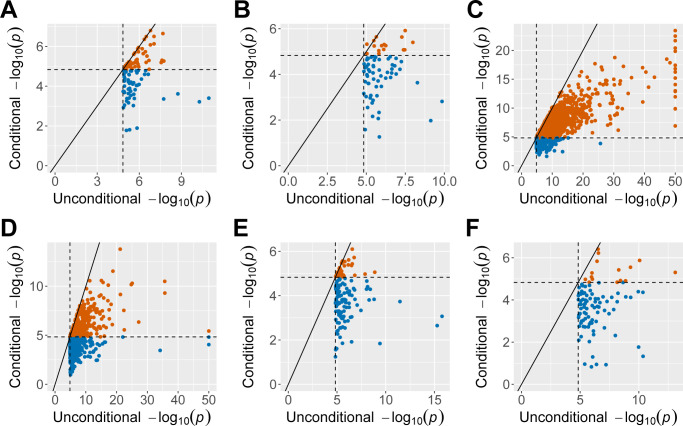
Comparison of finite-sample FiMAP p values from RaPID IBD segments before and after conditioning on GWAS tag variants in the testing window and flanking regions. (A) Waist circumference; (B) Hip circumference; (C) Standing height; (D) Sitting height; (E) Body mass index; (F) Body weight. IBD segments called by RaPID with length ≥ 3 cM were used in the FiMAP analysis. Dashed lines represented the Bonferroni-corrected significance level of 0.05/3,403 = 1.47 × 10^−5^. Only significant unconditional FiMAP p values were shown. P values < 1 × 10^−50^ for standing and sitting height were truncated at 1 × 10^−50^. FiMAP conditional p-values that no longer reach significance were shown in blue.

**Fig 6:**
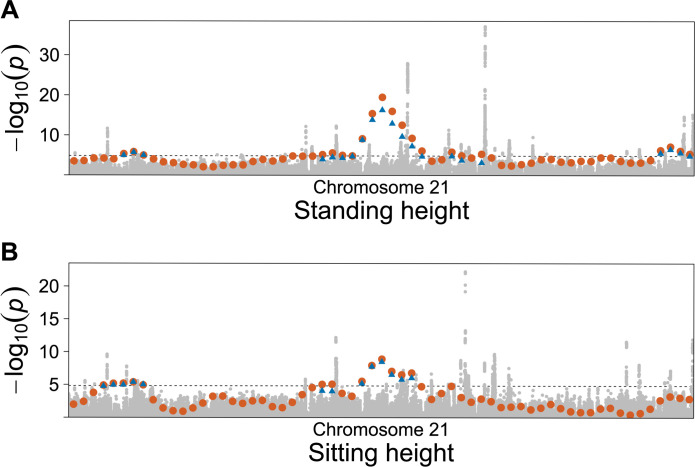
GWAS and finite-sample FiMAP p values from RaPID IBD segments on chromosome 21. (A) Standing height; (B) Sitting height. IBD segments called by RaPID with length ≥ 3 cM were used in the FiMAP analysis. GWAS p values were shown in grey and unconditional FiMAP p values were shown in orange. For windows with unconditional FiMAP p values < 0.05/3,403 = 1.47 × 10^−5^, conditional p values after adjusting for all tag variants in each conditional set were shown in blue triangles.

For each anthropometric trait from the UK Biobank, the FiMAP analysis using the RaPID IBD length cutoff 3 cM took 90 CPU hours with a maximum memory footprint scaling linearly with the number of nonzero entries in the local IBD matrix *Ψ*_*l*_. RaPID IBD segment calling in the UK Biobank incurred a one-time cost of 474 CPU hours. As a comparison, the GWAS of 89 million unfiltered imputed variants using GMMAT [44] took an average of 1,125 CPU hours on the same computing node for each anthropometric trait. **Table 2** shows that FiMAP using RaPID IBD segments with length ≥ 3 cM identified 1,587 significant 1 cM windows without any GWAS suggestive evidence as there were no imputed genetic variants with MAF ≥ 0.0001, imputation quality score ≥ 0.3, and GWAS single-variant test p-value < 1×10^−6^ in these regions. On the other hand, it also shows that 1,855 significant 1 cM windows in FiMAP results had at least one such GWAS tag variant in the same test region. Interestingly, 2,131 out of 3,442 (62%) 1 cM windows remained significant even after a very stringent conditional analysis by adjusting for all tag variants in the conditional set, selected as the most significant variants with GWAS single-variant test p-value < 1×10^−6^ and physical distance > 100kb in the ± 3 cM flanking regions (a total length of 7 cM), suggesting that IBD mapping provided complementary association evidence missed by a traditional GWAS approach. **Fig 5** shows that for windows with a significant unconditional FiMAP p-value, conditional p-values were generally larger (less significant) than unconditional p-values as expected, for all 6 anthropometric traits. Of note, we identified a 6 cM region (29–35 cM) on chromosome 21 associated with both standing height and sitting height using RaPID IBD segments with length ≥ 3 cM (**Fig 6**, minimum p values after conditioning on all tag variants in each 7 cM region: standing height 5.1 × 10^−17^, sitting height 3.3 × 10^−9^).

**Table 2:** Numbers of significant 1 cM windows in UK Biobank unconditional and conditional IBD mapping for 6 anthropometric traits.

Trait	Unconditional	No GWAS suggestive evidence	Conditional
Waist circumference	99	48 (48%)	46 (46%)
Hip circumference	74	35 (47%)	16 (22%)
Standing height	2,189	1,051 (48%)	1,652 (75%)
Sitting height	842	359 (43%)	364 (43%)
Body mass index	138	63 (46%)	38 (28%)
Body weight	100	31 (31%)	15 (15%)

IBD segments called by RaPID with length ≥ 3 cM were used in the FiMAP analysis. Statistical significance was defined as finite-sample p value < 0.05/3,403 = 1.47 × 10^−5^ for both unconditional and conditional FiMAP. No GWAS suggestive evidence was defined as a significant 1 cM unconditional FiMAP test region with no imputed genetic variants with MAF ≥ 0.0001, imputation quality score ≥ 0.3, and GWAS single-variant test p-value < 1×10^−6^ in the same 1 cM test region. Conditional FiMAP tests were performed after adjusting for all tag variants in each conditional set, selected as the most significant variants with MAF ≥ 0.0001, imputation quality score ≥ 0.3, GWAS single-variant test p-value < 1×10^−6^, and physical distance > 100kb in the ± 3 cM flanking regions (a total length of 7 cM) from GWAS.

For four anthropometric traits related to body fat distribution or weight (waist circumference, hip circumference, body mass index, and body weight), we found highly significant FiMAP p values near the *FTO* region on chromosome 16, using RaPID IBD segments with length ≥ 3 cM. However, none of the conditional p values remained significant after adjusting for GWAS tag variants in this region, suggesting that FiMAP did not provide additional association evidence beyond GWAS in this case (**S3 Fig**).

As both RaPID and FiMAP rely on random algorithms, we also used a different random number seed in RaPID IBD segment calling, as well as FiMAP IBD mapping, to investigate the numerical stability of our findings. **S4** and **S5 Figs** show that FiMAP p values were highly stable with respect to different random number seeds used in IBD segment calling by RaPID (minimum Spearman’s *ρ* = 0.966) and IBD mapping (minimum Spearman’s *ρ* = 0.989). These results suggested that our top findings were robust against different random number seeds used in IBD segment calling and/or IBD mapping.

## Discussion

In this work, we have developed FiMAP to conduct fast IBD mapping analysis that scales linearly with the sample size and applied it to the UK Biobank data. Compared to traditional GWAS approaches that utilize only unphased genotypes, IBD mapping with the phase information offers a distinct perspective into the genetic architecture of complex traits by leveraging the genotype similarity in a specific genomic region. The heuristics that if a genomic region is associated with a complex trait, then individuals with greater genotype similarity are also expected to show greater phenotype similarity, have led to the development of linkage analysis methods for family data, including the variance component linkage analysis. However, such methods have not previously been widely applied to biobank-scale population-based cohorts, due to computational challenges.

FiMAP is an accurate and computationally efficient IBD mapping method. It leverages a random matrix to approximate the null distribution of the variance component test statistic in quadratic form. We have found in the UK Biobank data analysis that different random number seeds used in either IBD segment calling by RaPID or IBD mapping by FiMAP had minimal impact on the top association findings, as the p values were highly consistent regardless of the different random number seeds. In addition, our simulation results using IBD segments from two different IBD callers RaPID and hap-IBD were also highly concordant given the same length cutoff, suggesting that FiMAP was robust with respect to which IBD caller was used.

Our simulation studies showed that FiMAP is often more powerful using IBD segments with shorter length cutoffs. However, there is also a tradeoff between statistical power and computational efficiency. With an IBD length cutoff of 10 cM, FiMAP was very computationally efficient, but also had the lowest power in our simulation settings. With an IBD length cutoff of 3 cM, FiMAP was often more powerful than using cutoffs of 5 cM and 10 cM, but the run time also dramatically increased. With even shorter IBD length cutoffs (e.g., 2 cM), a much larger number of IBD segments are expected to be called (see Equation 14 of Palamara *et al*. [5]), which may decrease the sparsity of local IBD matrices and lead to a substantial increase in both the run time and memory footprint. In our real data analysis, we chose an IBD length of 3 cM to balance between power and computational efficiency.

In general, the power of IBD mapping depends on the number of IBD segments each sample has in a particular genomic window, which grows quadratically with N. For example, with an IBD length cutoff of 3 cM for RaPID, the median number of non-zero off-diagonal elements in local IBD matrices was 60.4 × N in the full sample (N = 487,252), compared to 0.124 × N in a random subset of N = 1,000 individuals. Therefore, we expect FiMAP to have much greater statistical power in biobank-scale cohorts, although the actual power may also depend on other factors such as the genetic architecture of the trait, as well as the degree of relatedness in the samples. On the other hand, when N is much larger than the size of UK Biobank (in a somewhat distant future), the local IBD matrix may not be sparse enough and new method than FiMAP may be needed for efficient IBD mapping.

Surprisingly, asymptotic p values from FiMAP are not well calibrated even with the sample sizes of the UK Biobank. Although the *N*×*N* local IBD matrices are large in biobank-scale cohorts, they are often sparse with a limited number of non-zero off-diagonal elements. As a result, the asymptotic p values from FiMAP are extremely conservative in the tail for sparse local IBD matrices. In contrast, our simulation studies under the null hypothesis of no genetic association showed that finite-sample FiMAP p values followed a uniform distribution, after accounting for the variability in estimating the residual variance parameter. Therefore, we recommend the use of finite-sample FiMAP p values for all analyses.

FiMAP, or IBD mapping in general, has several advantages compared to traditional GWAS approaches on unphased genotypes. It may better tag rare causal variants, especially when they are not directly genotyped, which is often the case for commonly used genotyping arrays. Although it is unlikely that rare causal variants are not genotyped using the whole genome sequencing technology, as the IBD calling algorithm such as RaPID tolerates mismatches, FiMAP is more robust to genotyping or sequencing errors since each individual genetic variant would not dramatically change the IBD segment calling. Moreover, the phasing information is usually ignored in traditional GWAS approaches. Our conditional analysis for UK Biobank anthropometric traits showed that many 1 cM windows identified by FiMAP remained significant after adjusting for all GWAS tag variants in the testing window and flanking regions, suggesting that these associations could not be fully explained by unphased genotypes, and therefore haplotype effects might play an important role in the genetic architecture of complex traits. With recent advances in fully phased human genome assembly from long-read sequencing data [60,61], more haplotype effects are expected to be identified for complex traits in the future. As an alternative to IBD mapping, haplotype-based association tests [62–65] have been proposed and applied, however, scaling these methods to biobank-scale cohorts remains a computational challenge.

FiMAP also has a few limitations. It tests a few thousand 1 cM windows on the human genome, and therefore requires a much less stringent significance level than traditional GWAS approaches which test millions of genetic variants. However, the major limitation of FiMAP is the low resolution of association signals. Follow-up analysis is often needed to identify causal variants or interpret these association findings. On the other hand, we note that IBD mapping has the potential of achieving a higher resolution than admixture mapping as IBD segments tend to be shorter than local ancestry tracts in recently admixed populations. Also, currently FiMAP can only be applied to quantitative traits in a linear mixed model framework, and future work includes extension to binary traits using logistic mixed models, as well as survival traits using Cox mixed models. Nevertheless, FiMAP empowers computationally efficient IBD mapping for complex traits using variance component linkage analysis models with unprecedented sample sizes from biobank-scale cohorts.

## Supporting information

S1 FigQuantile-quantile plot of finite-sample and asymptotic IBD mapping variance component test p values under the null hypothesis.Results from the dense *N*×*N* projection matrix $\hat{P}$ estimated from the null model and the FiMAP algorithm with the number of random vectors *B* = 100, 1,000 or 10,000 were shown. A random subset of *N* = 10,000 samples were taken from the UK Biobank, and 3,403 random *N*×*N* local IBD matrices with 10,000, 100,000 and 1 million non-zero off-diagonal elements were simulated under the null hypothesis of no association. Finite-sample p values from local IBD matrices with (A) 10,000, (B) 100,000, or (C) 1 million non-zero off-diagonal elements, and asymptotic p values from local IBD matrices with (D) 10,000, (E) 100,000, or (F) 1 million non-zero off-diagonal elements were plotted against expected p values from a uniform distribution.(TIF)

S2 FigScatter plot of finite-sample and asymptotic IBD mapping variance component test p values under the null hypothesis.Results from the dense *N*×*N* projection matrix $\hat{P}$ estimated from the null model and the FiMAP algorithm with the number of random vectors *B* = 100, 1,000 or 10,000 were compared. A random subset of *N* = 10,000 samples were taken from the UK Biobank, and 3,403 random *N*×*N* local IBD matrices with 10,000, 100,000 and 1 million non-zero off-diagonal elements were simulated under the null hypothesis of no association. Finite-sample FiMAP p values from local IBD matrices with (A) 10,000, (B) 100,000, or (C) 1 million non-zero off-diagonal elements were plotted against finite-sample variance component test p values using the dense *N*×*N* projection matrix $\hat{P}$. Asymptotic variance component test p values using the dense *N*×*N* projection matrix $\hat{P}$, from local IBD matrices with (D) 10,000, (E) 100,000, or (F) 1 million non-zero off-diagonal elements were plotted against finite-sample variance component test p values using the dense *N*×*N* projection matrix $\hat{P}$. Asymptotic FiMAP p values from local IBD matrices with (G) 10,000, (H) 100,000, or (I) 1 million non-zero off-diagonal elements were plotted against asymptotic variance component test p values using the dense *N*×*N* projection matrix $\hat{P}$.(TIF)

S3 Fig GWAS and finite-sample FiMAP p values from RaPID IBD segments on chromosome 16. (A) Waist circumference; (B) Hip circumference; (C) Body mass index; (D) Body weight. IBD segments called by RaPID with length ≥ 3 cM were used in the FiMAP analysis. GWAS p values were shown in grey and unconditional FiMAP p values were shown in orange. GWAS p values < 1 × 10^−25^ were truncated at 1 × 10^−25^. For windows with unconditional FiMAP p values < 0.05/3,403 = 1.47 × 10^−5^, conditional p values after adjusting for all tag variants in each conditional set were shown in blue triangles.
(TIF)

S4 Fig Comparison of finite-sample FiMAP p values using two separate RaPID IBD segment calls with different random number seeds. (A) Waist circumference; (B) Hip circumference; (C) Standing height; (D) Sitting height; (E) Body mass index; (F) Body weight. IBD segments called by RaPID with length ≥ 3 cM were used in the FiMAP analysis. Black dashed lines represented the Bonferroni-corrected significance level of 0.05/3,403 = 1.47 × 10^−5^. P values < 1 × 10^−50^ for standing and sitting height were truncated at 1 × 10^−50^. Spearman’s rank correlation coefficient was calculated for p values from two separate runs.
(TIF)

S5 Fig Comparison of finite-sample FiMAP p values using two random matrices with different random number seeds. (A) Waist circumference; (B) Hip circumference; (C) Standing height; (D) Sitting height; (E) Body mass index; (F) Body weight. IBD segments called by RaPID with length ≥ 3 cM were used in the FiMAP analysis. Black dashed lines represented the Bonferroni-corrected significance level of 0.05/3,403 = 1.47 × 10^−5^. P values < 1 × 10^−50^ for standing and sitting height were truncated at 1 × 10^−50^. Spearman’s rank correlation coefficient was calculated for p values from two separate runs.
(TIF)

S1 Table FiMAP analysis results for waist circumference in the UK Biobank. Start and end positions in centimorgan (cM) are shown, along with the number of non-zero off-diagonal elements in each N × N local IBD matrix and finite-sample p values (N = 407,872).
(XLSX)

S2 Table FiMAP analysis results for hip circumference in the UK Biobank. Start and end positions in centimorgan (cM) are shown, along with the number of non-zero off-diagonal elements in each N × N local IBD matrix and finite-sample p values (N = 407,827).
(XLSX)

S3 Table FiMAP analysis results for standing height in the UK Biobank. Start and end positions in centimorgan (cM) are shown, along with the number of non-zero off-diagonal elements in each N × N local IBD matrix and finite-sample p values (N = 407,681).
(XLSX)

S4 Table FiMAP analysis results for sitting height in the UK Biobank. Start and end positions in centimorgan (cM) are shown, along with the number of non-zero off-diagonal elements in each N × N local IBD matrix and finite-sample p values (N = 407,323).
(XLSX)

S5 Table FiMAP analysis results for body mass index in the UK Biobank. Start and end positions in centimorgan (cM) are shown, along with the number of non-zero off-diagonal elements in each N × N local IBD matrix and finite-sample p values (N = 407,255).
(XLSX)

S6 Table FiMAP analysis results for body weight in the UK Biobank. Start and end positions in centimorgan (cM) are shown, along with the number of non-zero off-diagonal elements in each N × N local IBD matrix and finite-sample p values (N = 407,400).
(XLSX)
