# Supplementary figures and images for: FiMAP: A fast identity-by-descent mapping test for biobank-scale cohorts
Source: PLoS Genet. 2023 Dec 1;19(12):e1011057. doi: 10.1371/journal.pgen.1011057 (PMC10718418; doi:10.1371/journal.pgen.1011057)

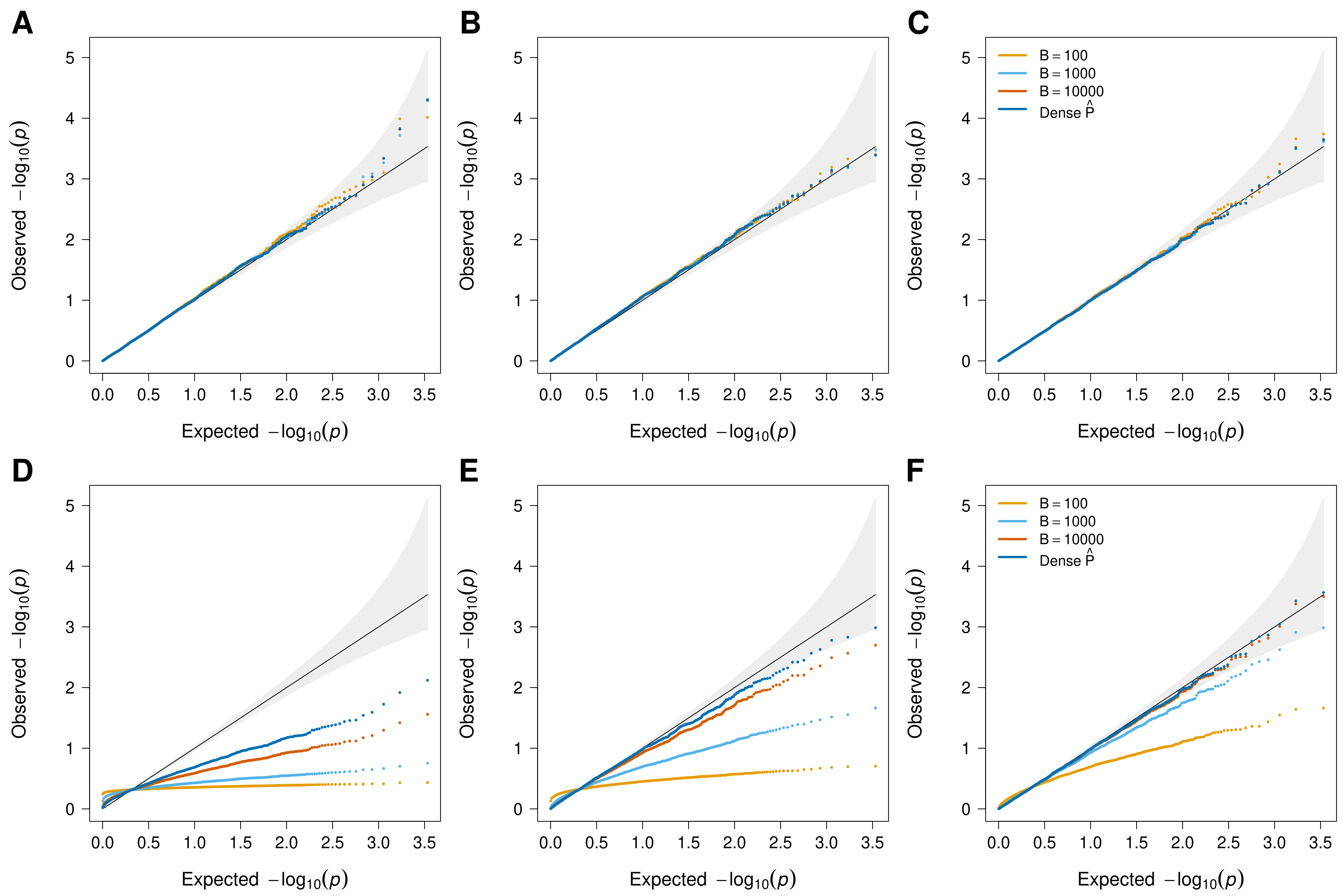

Supplement: S1 Fig — Results from the dense N×N projection matrix P^ estimated from the null model and the FiMAP algorithm with the number of random vectors B = 100, 1,000 or 10,000 were shown. A random subset of N = 10,000 samples were taken from the UK Biobank, and 3,403 random N×N local IBD matrices with 10,000, 100,000 and 1 million non-zero off-diagonal elements were simulated under the null hypothesis of no association. Finite-sample p values from local IBD matrices with (A) 10,000, (B) 100,000, or (C) 1 million non-zero off-diagonal elements, and asymptotic p values from local IBD matrices with (D) 10,000, (E) 100,000, or (F) 1 million non-zero off-diagonal elements were plotted against expected p values from a uniform distribution. (TIF) [file pgen.1011057.s001.tif]

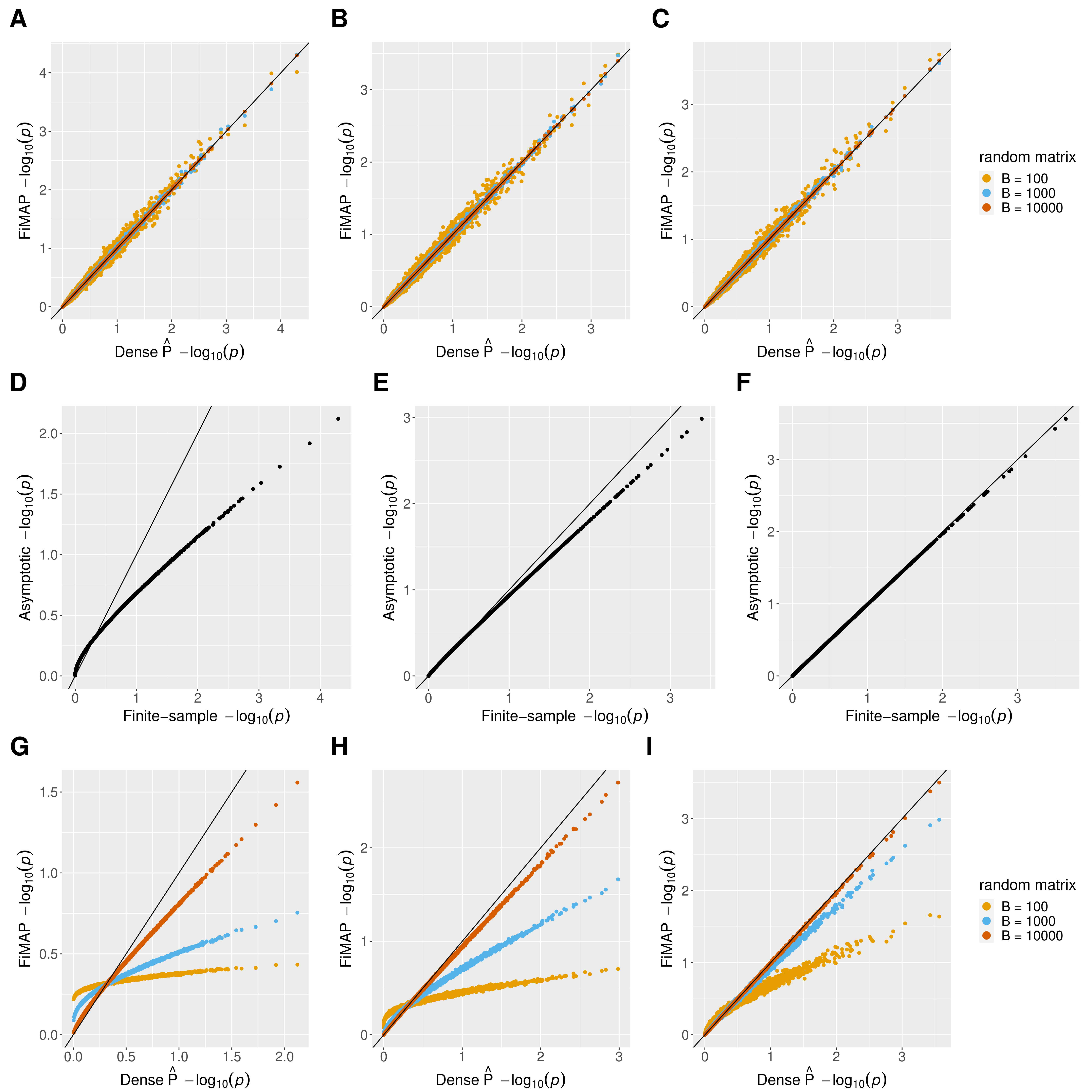

Supplement: S2 Fig — Results from the dense N×N projection matrix P^ estimated from the null model and the FiMAP algorithm with the number of random vectors B = 100, 1,000 or 10,000 were compared. A random subset of N = 10,000 samples were taken from the UK Biobank, and 3,403 random N×N local IBD matrices with 10,000, 100,000 and 1 million non-zero off-diagonal elements were simulated under the null hypothesis of no association. Finite-sample FiMAP p values from local IBD matrices with (A) 10,000, (B) 100,000, or (C) 1 million non-zero off-diagonal elements were plotted against finite-sample variance component test p values using the dense N×N projection matrix P^. Asymptotic variance component test p values using the dense N×N projection matrix P^, from local IBD matrices with (D) 10,000, (E) 100,000, or (F) 1 million non-zero off-diagonal elements were plotted against finite-sample variance component test p values using the dense N×N projection matrix P^. Asymptotic FiMAP p values from local IBD matrices with (G) 10,000, (H) 100,000, or (I) 1 million non-zero off-diagonal elements were plotted against asymptotic variance component test p values using the dense N×N projection matrix P^. (TIF) [file pgen.1011057.s002.tif]

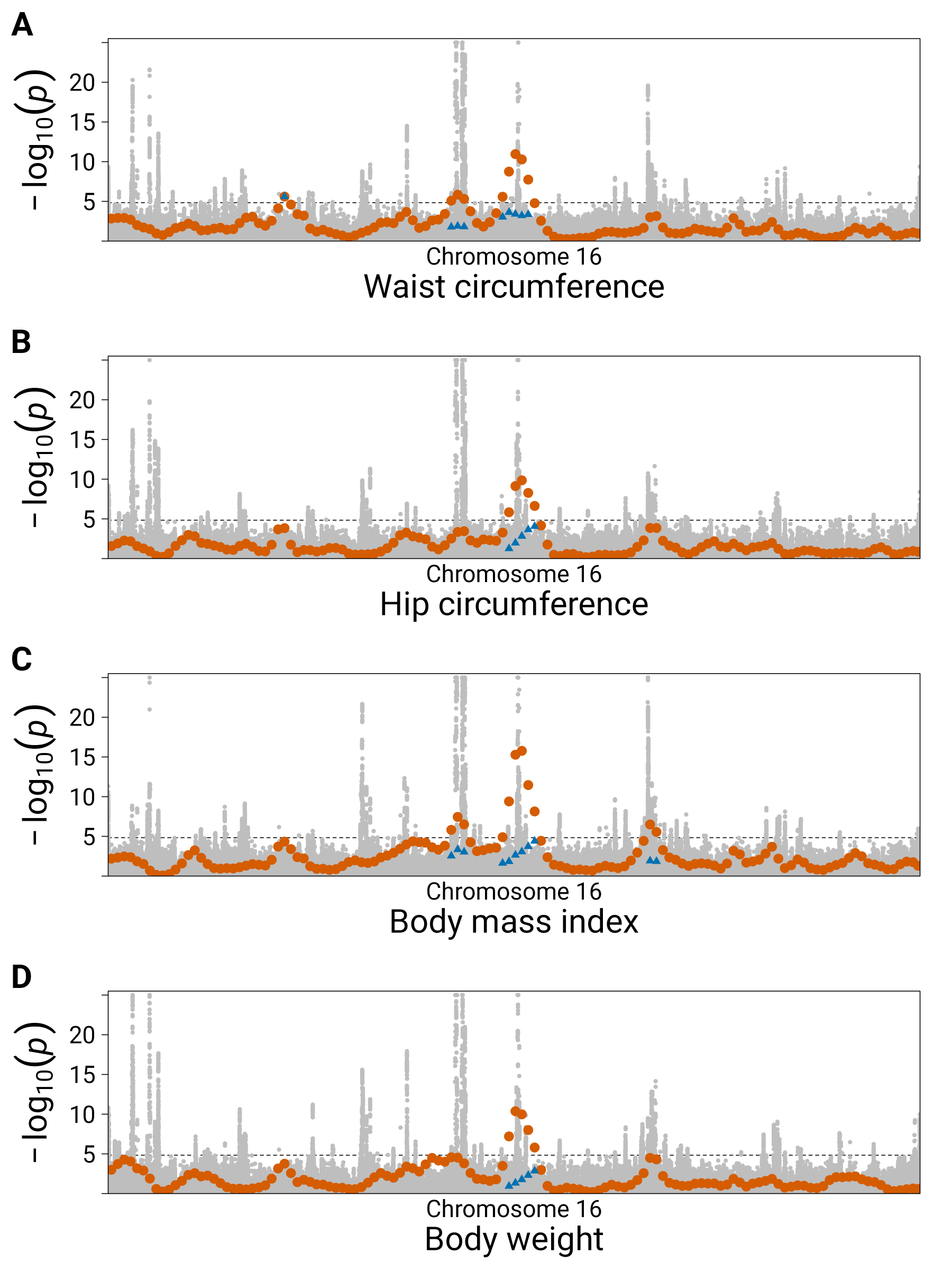

Supplement: S3 Fig — (A) Waist circumference; (B) Hip circumference; (C) Body mass index; (D) Body weight. IBD segments called by RaPID with length ≥ 3 cM were used in the FiMAP analysis. GWAS p values were shown in grey and unconditional FiMAP p values were shown in orange. GWAS p values < 1 × 10^−25^ were truncated at 1 × 10^−25^. For windows with unconditional FiMAP p values < 0.05/3,403 = 1.47 × 10^−5^, conditional p values after adjusting for all tag variants in each conditional set were shown in blue triangles. (TIF) [file pgen.1011057.s003.tif]

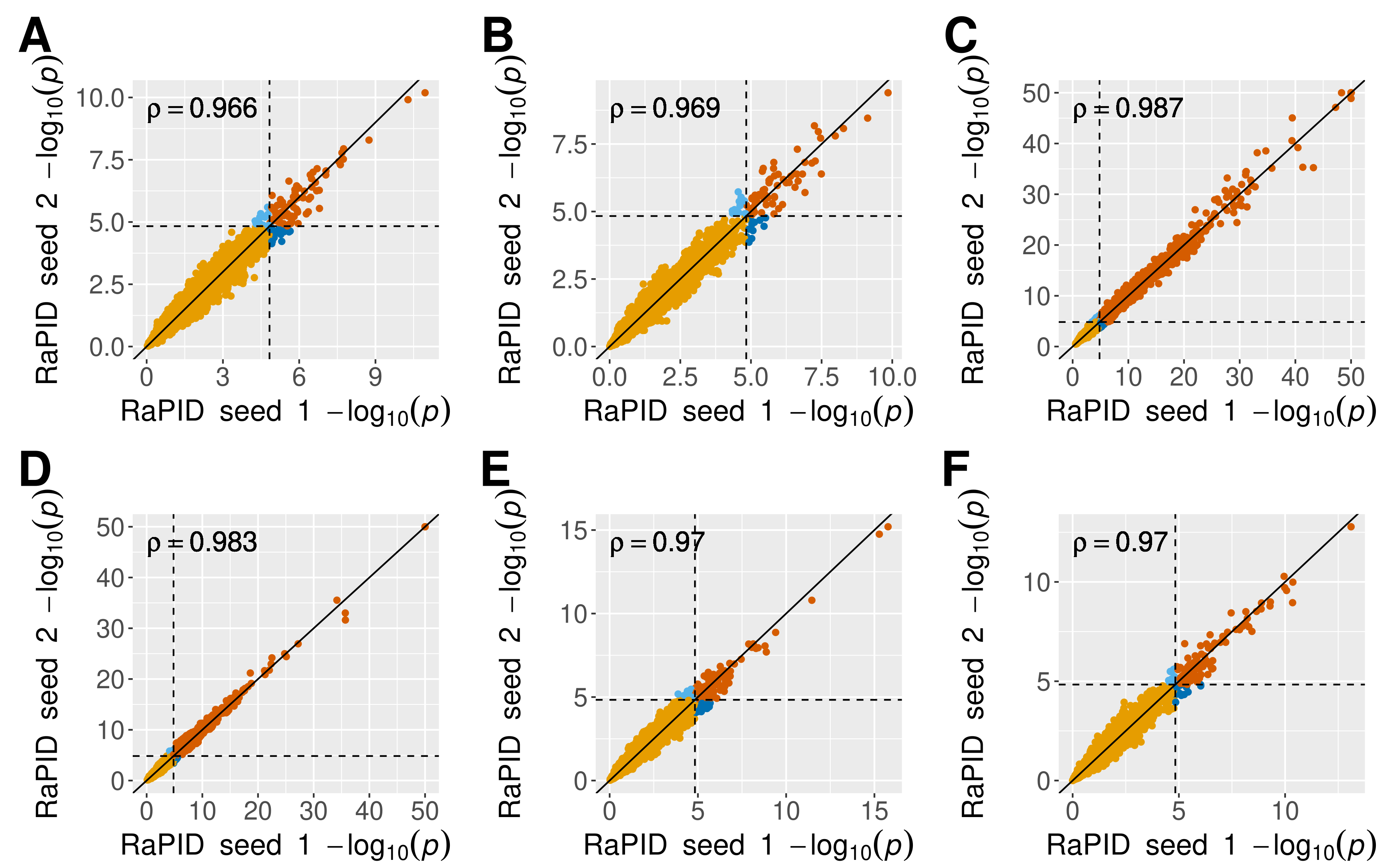

Supplement: S4 Fig — (A) Waist circumference; (B) Hip circumference; (C) Standing height; (D) Sitting height; (E) Body mass index; (F) Body weight. IBD segments called by RaPID with length ≥ 3 cM were used in the FiMAP analysis. Black dashed lines represented the Bonferroni-corrected significance level of 0.05/3,403 = 1.47 × 10^−5^. P values < 1 × 10^−50^ for standing and sitting height were truncated at 1 × 10^−50^. Spearman’s rank correlation coefficient was calculated for p values from two separate runs. (TIF) [file pgen.1011057.s004.tif]

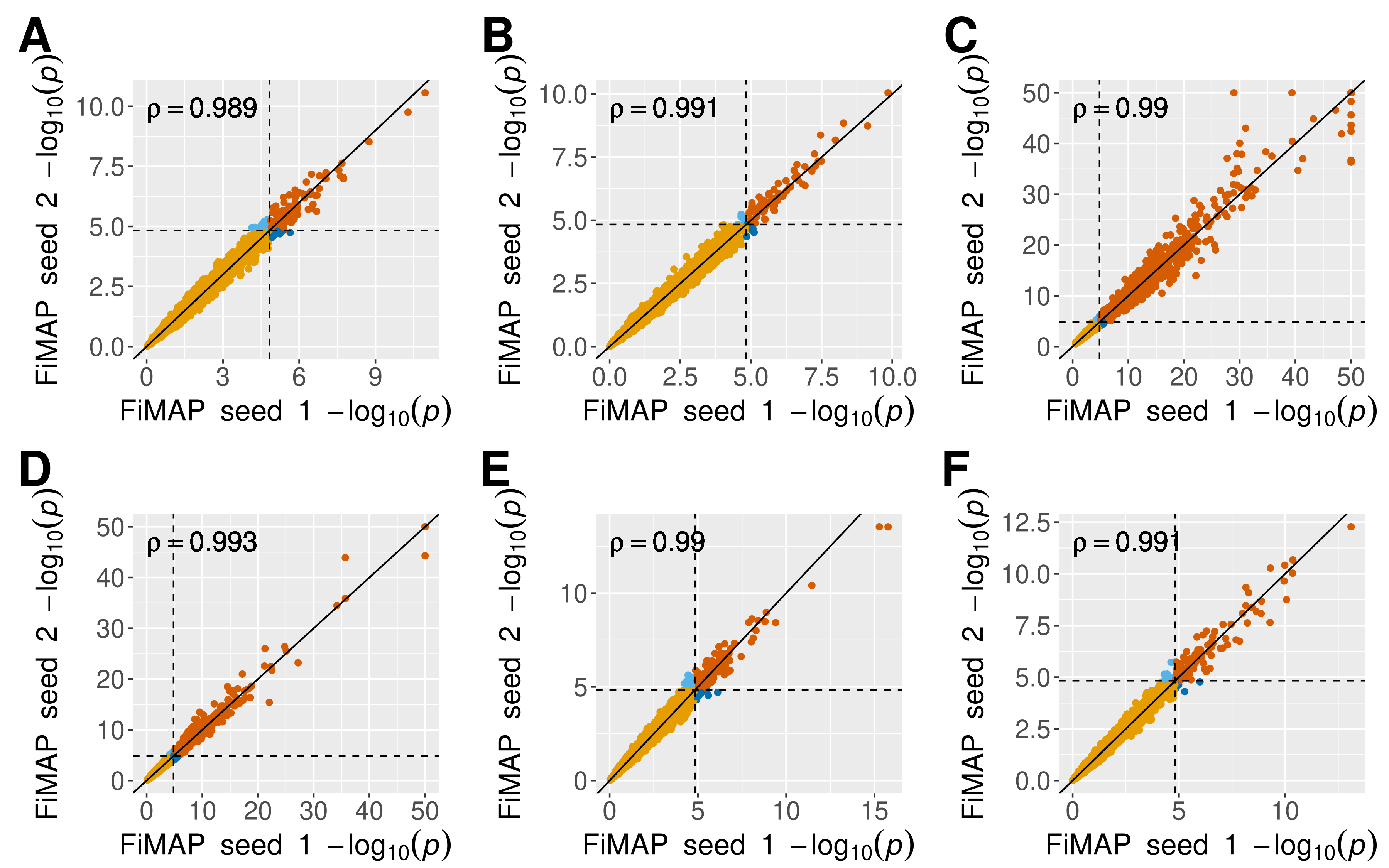

Supplement: S5 Fig — (A) Waist circumference; (B) Hip circumference; (C) Standing height; (D) Sitting height; (E) Body mass index; (F) Body weight. IBD segments called by RaPID with length ≥ 3 cM were used in the FiMAP analysis. Black dashed lines represented the Bonferroni-corrected significance level of 0.05/3,403 = 1.47 × 10^−5^. P values < 1 × 10^−50^ for standing and sitting height were truncated at 1 × 10^−50^. Spearman’s rank correlation coefficient was calculated for p values from two separate runs. (TIF) [file pgen.1011057.s005.tif]
